# Pathobiology of ESKAPE Biofilms in implant infections: current understanding and implications for future therapeutic strategies

**DOI:** 10.3389/fcimb.2026.1750702

**Published:** 2026-03-09

**Authors:** Frangleena P. S., K. Suthindhiran

**Affiliations:** Marine Biotechnology and Bioproducts Laboratory, Department of Bio-Medical Sciences, School of Bio Sciences and Technology, VIT, Vellore, Tamil Nadu, India

**Keywords:** biomaterials, ESKAPE pathogens, implant-associated infection, implants, microbial biofilm, quorum sensing

## Abstract

In the modern era, the expanding demand for implants has transformed the healthcare system by restoring and enhancing the function of various biological structures, thereby increasing the patients’ quality of life. These include urinary catheters, dental, orthopedic, cardiovascular implants, and sutures designed to perform various functions. However, these devices are more prone to microbial attack, contributing to biofilm formation mainly caused by multidrug-resistant ESKAPE pathogens, thereby increasing the risk of implant-associated infections and implant failure. This review summarizes the diverse array of implants available on the market and their associated infections caused by biofilm-producing pathogens, with a particular emphasis on the ESKAPE pathogen. Specific keywords were used to conduct a literature review using Google Scholar, Web of Science, PubMed, and Scopus databases. The data were then screened and integrated to explore the underlying principles of biofilm formation, its consequences, diagnostic approaches, and therapeutic studies. Currently, various methods are employed to diagnose these infections, including culture-based methods (tissue swab, culture, sonication) and non-culture methods (Dithiothreitol, XTT (2,3-bis-(2-methoxy-4-nitro-5-sulfophenyl)-2H-tetrazolium-5-carboxanilide), Resazurin, BioTimer assays, and PCR). However, these studies indicate an increased difficulty in detecting infections caused by ESKAPE pathogens due to biofilm formation, highlighting the need for developing novel strategies. The recent advancements in the development of antimicrobial coatings, implant surface modifications, phage therapy, nanoparticles, antimicrobial peptides, and quorum-sensing inhibitors have shown promise in controlling these infections. Thus, these findings underscore the importance of research on innovative approaches and the development of infection-resistant implants, thereby reducing the clinical burden and improving patient outcomes.

## Graphical Abstract

**Figure:**
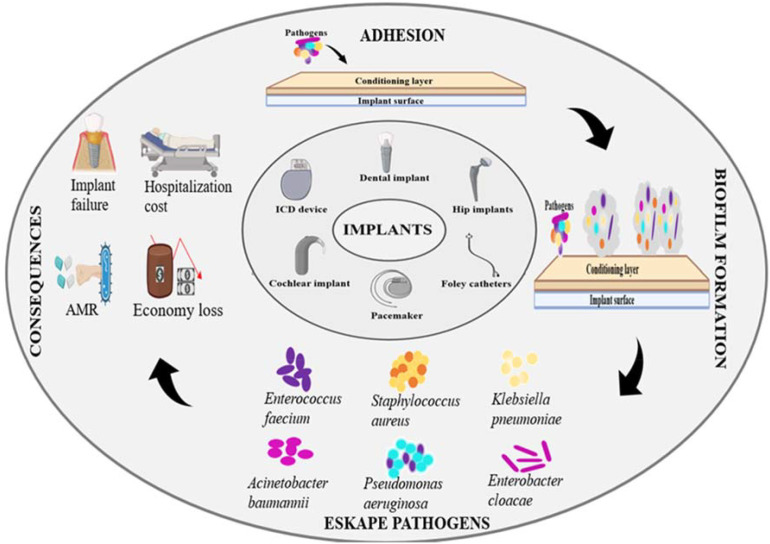

## Introduction

The introduction of implants has enhanced the efficiency and functionality of organs such as the heart, bones, and teeth. The primary function of these devices is to replace damaged parts, restore appearance, reduce discomfort, and improve the performance of various biological structures. Today, numerous types of implants are available on the market, including stents, urinary devices, pacemakers, sutures, contact lenses, dental implants, endotracheal tubes, fracture fixation devices, and others, where these are used for various medical uses such as being inserted into the urethra, jawbone, muscles, bones, and other body parts. Their production relies heavily on biomaterials, including metals, alloys, ceramics, polymers, and composites, which are selected for their mechanical strength, durability, and biocompatibility (Teo et al., 2016; Narayana and Srihari, 2019; Ramezani and Ripin, 2023).

Apart from their medicinal benefits, they provide an environment that favors the growth and colonization of numerous bacteria by interacting with the implant surface, which can lead to implant-associated infections (IAI). The colonization of bacteria such as *Staphylococcus* sp.*, Streptococcus* sp.*, Corynebacterium* sp.*, Cutibacterium acnes, Escherichia coli, Pseudomonas* sp.*, Klebsiella pneumoniae, Providencia stuartii, and others* on the surfaces of implanted medical devices made of inert materials and polymers is a significant contributing factor to IAI (Nikolaev and Plakunov, 2007; Dongari-Bagtzoglou, 2008; Kandi and Vadakedath, 2020). These infections are due to the formation of biofilms on implant surfaces, structured groups of sessile cells enclosed within an extracellular polymeric matrix that provides protection against antimicrobial agents, nutrient limitations, and various immune responses. However, quorum sensing plays a vital role in regulating the formation of biofilm by enabling bacteria to communicate with each other and to coordinate their response to external stimuli (Whitchurch et al., 2002; Lu et al., 2022; Tiwari, 2023).

The National Institutes of Health reports have indicated that up to 80% infections in humans due to microbes arise from biofilm formation, leading to implant infections (Nandakumar et al., 2013; Khatoon et al., 2018; Mishra et al., 2024). The proliferation of multidrug-resistant (MDR) microorganisms, particularly a group (*Enterococcus faecium, Staphylococcus aureus, Klebsiella pneumoniae, Acinetobacter baumannii, Pseudomonas aeruginosa*, and *Enterobacter* sp.), drives the formation of bacterial biofilms and the associated infections. Forming biofilms is a key mechanism by which drug-resistant and multidrug-resistant ESKAPE pathogens exhibit antimicrobial resistance. These multidrug-resistant (MDR) pathogens have developed antibiotic resistance, shielding biofilms from antimicrobial agents, leading to persistent infections that are difficult to eradicate. As these biofilms exhibit high collective resistance due to horizontal gene transfer and mutation rates, and are recognized as reservoirs for antibiotic-related genes (Lin et al., 2018; Uruén et al., 2020; Saini et al., 2024). The ESKAPE pathogens are considered the primary contributors to biofilm-associated infections and are responsible for nearly 40% of infections in intensive care units, according to records of the National Healthcare Safety Network (Bennett et al., 2023).

Recent therapeutical approaches such as phage-based therapies, nanomedicine-based interventions, and prophylactic strategies altering the implant surface have helped to address the challenges due to biofilm (Lu et al., 2022). The risks involved with IAI in patients remain, including loss of function, increased susceptibility to infections, tissue damage, and financial burden. Thus, there is a need to develop implants that are effective, biocompatible, infection-resistant, and long-lasting. This review focuses on implant types, biofilm formation on implants by ESKAPE pathogens, implant-associated infections, and existing methods for treating and preventing IAI, highlighting the need for developing biofilm-resistant implants.

## The historical progression and growth of implant technology

Dating back to ancient civilizations, implants were used to enhance the function of various body parts. Archaeological evidence indicates that, the Mayan population in the 3rd century AD employed bow drills to fill up tooth gaps and used carved stones to replace the lost teeth. The Phoenicians in the 3rd century AD and the Etruscans in the 5th century AD utilized gold wire and bands, respectively, to restore the functionality of the oral cavity. Additionally, the ancient Egyptians around 2500 BC used gold wire to improve the stability of teeth (Pasqualini and Pasqualini, 2009; Abraham, 2014). Metal-based dental implants were developed during World War II for dental restorative purposes and gained wider acceptance, leading to the establishment of modern implants. The first use of titanium dental implants was reported in 1965, which were used to replace lost teeth. Later, in 1992, ceramic implants were introduced, which demonstrated enhanced osseointegration capabilities in patients (Gaviria et al., 2014). The remarkable progress in biomedical technology has enabled the introduction of a diverse array of implant materials in the healthcare market, which have consequently enhanced the quality of life for patients.

## Typologies of medical implants

Implants are becoming increasingly valuable day by day due to their beneficial impact on the physiological functions in the patient. As a result, there are wide variety of implants being used in the medical field, including contact lenses, urinary catheters, sutures, stents, pacemakers, fracture fixation devices, dental implants, hip implants, breast implants, and endotracheal implants, which are inserted into various body parts, such as the eyes, urethra, skin, blood vessels, bones, and teeth. Therefore, numerous biomaterials like polymers, steel wire, silicone, latex, zirconia, titanium, and cobalt-chromium alloys are used for development of these implants (Narayana and Srihari, 2019; Ramezani and Ripin, 2023; AD et al., 2024). The widespread adoption of implants has revolutionized the medical field due to their improved performance. **Table 1** summarizes the various biomaterials used in the production of medical implants, along with their respective benefits and applications.

**Table 1:** Biomaterials used in various implants and their corresponding targets.

S.NO	Implants	Target	Materials	Uses	Benefits	References
1	Dental implants	Jawbone	Titanium, cobalt-chromium alloy, alumina, zirconia, stainless steel, bioglass	Reconstruct teeth,	Enhance osseo-integration	(Narayana and Srihari, 2019; Saha and Roy, 2023)
2	Urinary catheter	Urethra	Latex, silicone, nylon, Polyethylene terephthalate	Helps to empty the bladder, prevents leakage of urine	Minimize the risk of infection and kidney damage	(Feneley et al., 2015; Narayana and Srihari, 2019)
3	Contact lenses	Eyes	Polymethylmethacrylate, polyhydroethylmethacrylate, silicone	Vision corrections, appearance of eyes can be changed	Fix eye problems, improved vision	(Narayana and Srihari, 2019; Dosler et al., 2020)
4	Breast implants	Breast	Silicone	Augmentation and Reconstruction of breast	Enhance the size, correction of asymmetries	(del Pozo and Auba, 2015; Narayana and Srihari, 2019)
5	Sutures	Skin, tissues, organs	Nylon, silk, polydioxanone, steel wire, polypropylene	Help to close wound cuts and surgical incision	Faster healing and strength, reduce the risk of infections	(Narayana and Srihari, 2019)
6	Fracture fixation devices	bone	Polymers, titanium alloys, stainless steel, cobalt chromium-magnesium alloys	Fractured bones are repaired	Mechanical stability, proper alignment of bone	(Narayana and Srihari, 2019; Stanciu and Diaz-Amaya, 2022)
7	Hip/knee implants	Knee joint	Stainless steel, titanium alloy, cobalt-chromium alloys, polyethylene, ceramics	To replace hip/knee	Treatment of arthritis,	(Narayana and Srihari, 2019; Marsh and Newman, 2021)
8	Coronary stents	Coronary arteries	Cobalt-chromium, titanium alloy, Magnesium alloy	blockage in coronary arteries is reduced	Elimination of coronary dissection and vascular recoil	(Narayana and Srihari, 2019; MacNeill et al., 2023)
9	Cardiac pacemakers	Heart	Titanium alloy, polyurethane, silicone	Heartbeat regulation	Treatment heart blockage	(Narayana and Srihari, 2019; Dalia and Amr, 2024)
10	Mechanical heart valve	Heart	Titanium alloys, graphite, polyester, pyrolytic carbon, cobalt-chromium	Reduce valvular heart disease	Durable, increased survival rate	(Harris et al., 2015; Narayana and Srihari, 2019)

The table provides a structured overview of the diverse range of implantable devices currently available, emphasizing their role in enhancing patient quality of life. It details the target anatomical sites, the materials used in their construction, and the intended applications and benefits associated with each implant type.

The Food and Drug Administration (FDA) classifies medical implants into three primary categories based on safety considerations and regulatory oversight. Class I devices, such as elastic bandages and handheld surgical instruments, are subject to general controls, including registration, manufacturing, labelling, and provision of FDA information, without requiring rigorous scientific evaluation. Class II devices, predominantly utilized in orthopedic procedures, sutures, surgical drapes, and infusion pumps, exhibit more than minimal potential for harm and thus require specialized controls. Finally, Class III devices, which includes high-risk items such as intramedullary nails, cannulated screws, plates, external fixators, pedicle screws, and rods, necessitate both general and special controls to ensure their safety and efficacy (van Eck et al., 2009). According to reports from the FDA and estimates from Medtech Europe, there are more than 500,000 types of medical implants available worldwide. **Table 2** is the representation of the implants, which are grouped into transient, permanent, intracorporeal (intravascular and extravascular), and extracorporeal implants based on their level of intrusiveness into the body, integration into different anatomical structures, and duration of use (Arciola et al., 2018).

**Table 2:** Overview of biomedical implants and their examples.

Class	Classification	Example	Reference
Transient	Used for short period. Removed/ naturally degrades	Urinary catheters Biodegradable implant (Cardiac Pacing Devices, Scaffolds, Drug delivery implants, Orthopedic implants)	(Rüegg et al., 2019; Xia et al., 2025)
Permanent	Introduced either partially/ wholly into the body through surgically/ medically Long-term use	Pacemakers	(Joung, 2013)
Intracorporea Intravascular	Interact with coagulation factors and circulating blood cells	Intravascular gas exchange catheter Pacemakers Left ventricular assist devices (LVADs)	(Michael and Timmons, 2010; Oliva et al., 2021; Straube et al., 2022)
Intracorporeal Extravascular	Interact with surrounding tissue, interstitial fluid, and attracted phagocytes	Implantable cardioverter-defibrillators (ICDs) Intracorporeal pressure measurement devices Orthopedic Implants (hip, knee prostheses)	(Michael and Timmons, 2010; Oliva et al., 2021; Hashmi et al., 2023)
Extracorporeal	Devices that remain outside the body Short to intermediate-term support	Extracorporeal oxygenators	(Lim, 2006; Berthiaume et al., 2016)

This table categorizes biomedical implants into various classifications based on their long-term or short-term nature, as well as their interaction within or outside the body, with corresponding examples.

## Infections stemming from implanted medical devices

The adhesion and accumulation of microorganisms especially on the surfaces of biomaterials used in medical implants, including stainless steel, nylon, polymers, silicone, chromium, titanium alloy, and various other alloys are more susceptible to biofilm formation. This susceptibility to biofilm formation is attributed to the presence of diverse microorganisms, particularly the ESKAPE pathogens (Mukherjee et al., 2023).

A higher percentage of hospital-related complications and the mortality due to infection is mainly observed due to nosocomial infections, which are infections that arise due to close contact with infected patients and their environment (Hocevar et al., 2012). A significant proportion, approximately 80%, of known pathogenic microorganisms have been associated with infections related to a diverse array of implanted medical devices, including intravenous and urinary catheters, joint prostheses, penile implants, contact lenses, fracture fixation devices, cardiovascular and biliary stents, and other such implanted medical technologies (Ruellan et al., 2010; Ramasamy and Lee, 2016). Biofilms on medical devices facilitate the transmission of pathogens and contribute to the development of infections. **Table 3** outlines IAI resulting from biofilm formation and its associated consequences. Microorganisms such as *S. aureus* and *Staphylococcus epidermidis* are recognized as the key contributors to healthcare-associated infections, causing a significant proportion of infections associated with various medical implants. These bacteria account for 31-52% of infections associated with orthopedic prosthetics, 40-50% of infections related to prosthetic heart valves, 50-70% of catheter-associated biofilm infections, and 87% of systemic infections (Nikoomanzari et al., 2022).

**Table 3:** Implant-associated infection caused by biofilm and its clinical morbidities.

S.NO	Implants	Infection/disease	Side effects	References
1	Dental implant	Peri-implant mucositis, peri-implantitis	Dental implant failure, tissue damage, increased inflammation	(Dhir, 2013; Barão et al., 2022)
2	Sutures	Surgical site infections, chronic wound infections	Prolonged hospitalization, affect tissue, death, suture dehiscence	(Hrynyshyn et al., 2022)
3	Urinary implants	Catheter-associated urinary tract infections	Increased risk of recurrent infections, biofilm upregulate toxins, cause tissue damage	(Lila et al., 2023)
4	Fracture fixation devices	Surgical site infection	Infection to soft and bone tissue	(Kanakaris and Giannoudis, 2021)
5	Breast implants	Breast implant illness, capsular contracture, chronic peri-implant inflammation	Skin and hair changes, and fatigue	(Lee et al., 2020)
6	Contact lenses	Contact lens-associated corneal infections (Microbial keratitis, contact lens-related acute red eye, contact lens peripheral ulcer, and infiltrative keratitis)	Loss of vision	(Kackar et al., 2017)
7	Mechanical heart valve	Native valve endocarditis	Damage to valve endothelium	(Jamal et al., 2018)
8	Cardiac pacemakers	Pacemaker related infections (pacemaker pocket infection)	Occurrence of endocarditis, high morbidity	(Paula et al., 2011)
9	Hip/knee implant	Prosthetic joint infections	Osteomyelitis, aging population and obesity epidemic	(Peel, 2019; Caldara et al., 2022)
10	Coronary stents	Coronary stent infection	pericarditis, myocardial infarction, myocardial rupture, and coronary aneurysm rupture	(Akhmetzhan et al., 2023)

Implants are widely used to enhance patient health and quality of life; however, they can also serve as ideal surfaces for bacterial colonization. This often results in the formation of resilient biofilms, which are a major cause of implant-associated infections. The table outlines various implant types, the specific infections commonly linked to biofilm formation on their surfaces, and the associated adverse effects on patient health, such as inflammation, tissue damage, and implant failure.

Urinary catheters: The colonization of bacteria on the periurethral skin facilitates in migration of bacteria into the bladder and causes biofilm on indwelling catheters (Stickler, 2008). Bacteria raise urine pH by promoting the development of struvite biofilms within catheters (Neethirajan et al., 2014).

Orthopedic implants: About 15% of hip implant failures related to infections require revision surgery to replace the implant (Bozic et al., 2009), causing inflammation and tissue damage. Techniques, including the modification of the surface textures of orthopedic implants through sintering (Gahlert et al., 2007), sandblasting (Grassi et al., 2007), plasma spraying can enhance their resistance to biofilm formation.

Joint prostheses: Implant failure owing to aseptic loosening is increasingly associated with underlying biofilm-driven infections. Infections of prosthetic joints by *S. epidermidis* or *C. acnes* can lead to severe complications and heightened mortality rates in patients following joint replacement procedures (del Pozo and Auba, 2015).

## Medical implants in India: usage trends and infections

In India, the use of implants has increased significantly due to population aging, rising chronic infections, and emerging technologies. Chronic infections such as diabetes, cardiovascular disease, and musculoskeletal disorders elevated the use of implants, including pacemakers, stents, and joint replacements, in the aging population. According to IMARC Group reports, the Indian implants market is valued at USD 115.4 billion in 2024 and is expected to reach USD 189.6 billion by 2033. Analyses of the Indian implant market indicate that orthopedic conditions such as osteoarthritis, fractures, and degenerative bone diseases are highly prevalent, contributing to increased demand for orthopedic implants (IMARC, 2024). The Reed Intelligence reports indicate that the Indian orthopedic implants market reached USD 1,018.13 million in 2024 and is projected to reach USD 1,629.47 million by 2033 (Reed Intelligence, 2024). In India, implant-associated infection affects nearly 6% of orthopedic implants, leading to economic loss to the patient (Sarkar et al., 2024). Orthopedic IAI are commonly due to *S. aureus*, and other risk factors include obesity, smoking, and longer surgery duration, with a reported prevalence of 25.7% (Kiran et al., 2023). Management of orthopedic IAI is challenging, as inappropriate antibiotic use can contribute to antimicrobial resistance and delay treatment outcomes, thereby resulting in prolonged hospitalization, increased morbidity, and an economic burden in patients (Shakthi and Venkatesha, 2023). Surgical site infection rates in India range from 1.6% to 38% by area, with *S. aureus* as the predominant pathogen. In this study, the incidence of infection was 7.6%, which is low compared to India’s highest reported figures but remained higher than that reported in high- and middle-income countries (Skender et al., 2022).

A recent study reported that the incidence of central line-associated bloodstream infections (CLABSI) was higher than in developed countries, and the associated pathogens were predominantly multidrug-resistant, such as *Acinetobacter* sp. (22%), followed by *K. pneumoniae* (16%) and *Enterobacter aerogenes* (16%) (Maqbool and Sharma, 2023). In India, almost 37,000 cardiac implanted electronic devices (CIEDs) were sold during the survey year, according to Eucomed data. This survey also highlights a marked gender imbalance, a gap that is even more pronounced for costly devices such as Implantable Cardioverter-Defibrillator (ICD) and cardiac resynchronization therapy (CRT), and where men receive the majority of CIED implants. Despite indications that women with dilated cardiomyopathy frequently benefit more from CRT, women are particularly disadvantaged in getting these treatments in a system with little government financing and insurance coverage (Shenthar et al., 2016).

## Microorganisms in biofilm establishment

Biofilm on implants is formed by various bacteria, including both Gram-positive species such as *Enterococcus faecalis, S. aureus, S. epidermidis*, and *Streptococcus viridans*, as well as Gram-negative species like *E. coli, K. pneumoniae, Proteus mirabilis*, and *P. aeruginosa*. The predominant organisms involved in biofilm formation are catalogued in **Table 4**. These pathogens can attach and form biofilms at the implantation site and on the implant device, leading to adverse outcomes such as implant failure, tissue damage, and associated infections (Veerachamy et al., 2014). Besides the bacteria previously mentioned, the ESKAPE pathogens has emerged as a major concern, causing persistent infections and leading to higher mortality rates (Tiwari, 2023).

**Table 4:** Major biofilm forming microbial species.

S.NO	Implants	Microorganisms	References
1	Dental implant	*S. viridans, Streptococcus mitis, Streptococcus oralis, Actinomyces* sp.*, Strptococcus mutans, Streptococcus sobrinus, Fusobacterium nucleatum, P. gingivalis, P. intermedia*	(Dhir, 2013)
2	Sutures	*S. aureus, Enterococcus* sp.*, E. coli, Streptococcus pyogenes*	(Hrynyshyn et al., 2022)
3	Urinary implants	*E. faecalis, P. aeruginosa, E. coli, P. mirabilis, S. aureus, K. pneumoniae, S. epidermidis, A. baumannii*	(Tenke et al., 2012; Lila et al., 2023)
4	Fracture fixation devices	*S. aureus, S. epidermidis, *coagulase-negative*Staphylococcus, S. viridans, E. faecalis, E. coli, K. pneumoniae, C. acnes, Peptostreptococci, P. mirabilis, A. baumanii*, and *P. aeruginosa.*	(Kanakaris and Giannoudis, 2021)
5	Breast implants	*P. acnes, S. epidermidis*	(Lee et al., 2020)
6	Contact lenses	*P. aeruginosa, Serratia* sp.*, S. aureus*,	(Bispo et al., 2015)
7	Mechanical heart valve	*Streptococci*, *Enterococci, S. epidermidis, S. aureus*	(Jamal et al., 2018; Choudhary et al., 2020)
8	Cardiac pacemakers	*S. aureus* and *S. epidermidis*	(Abdullah et al., 2021)
9	Hip/knee implant	*S. aureus, Staphylococcus lugdunensis*, *S. epidermidis*, Gram negative bacteria, *P. acnes*	(Caldara et al., 2022)
10	Coronary stents	*P. aeruginosa* and *S. epidermidis*	(Akhmetzhan et al., 2023)

This table provides an overview of key microbial species involved in biofilm formation, particularly on biomedical implants. These microbes have the potential to adhere to various surfaces, construct complex communities, and thereby develop resistance mechanisms against both antibiotics and the host's immune defenses.

## Biofilm structure and composition

**Figure 1:**
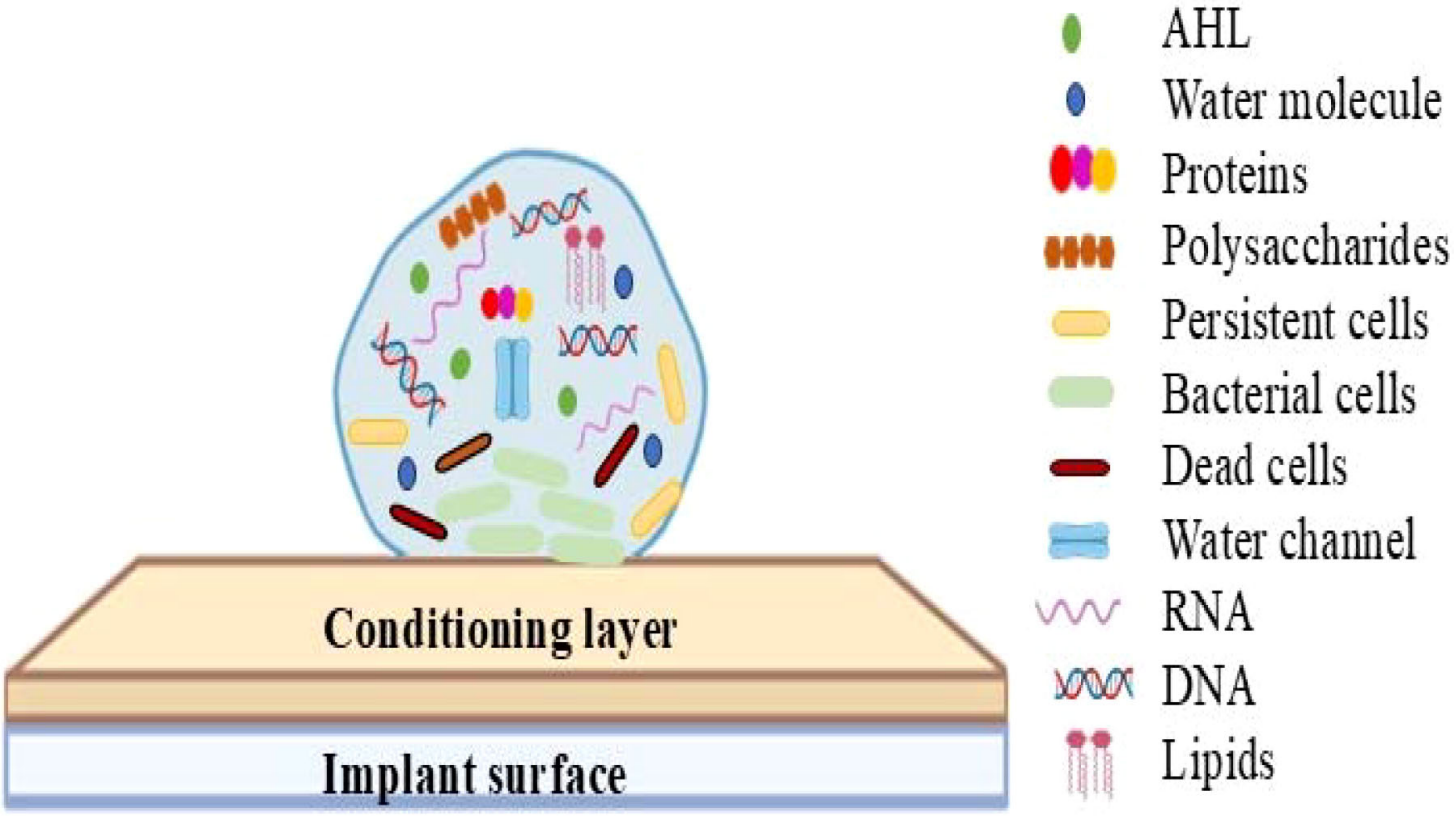
A schematic diagram illustrating the core components and structural organization of bacterial biofilms. This illustration represents a fully developed biofilm adhered to an implant surface, highlighting its structural components, including polysaccharides, DNA, RNA, lipids, water channels, quorum sensing molecules, proteins, and bacterial cells, all integrated within an extracellular polymeric substance matrix.

The microbial colonization on the implant surface forms a biofilm composed of a diverse group of microorganisms capable of producing extracellular polymeric substances (EPS) that protect the bacterial community from external forces. Biofilm is primarily made up of 90% water and 10% microbial mass. The biofilm matrix is composed of polysaccharides (EPS), which constitute 50-90% of the total organic components (Donlan, 2002; Abdelaziz et al., 2025) as illustrated in **Figure 1**. This matrix forms a thick, mesh-like structure, where the polysaccharide sequences with hydroxyl groups interact with each other, enhancing their mechanical strength. Calcium (Ca^2+^) and magnesium (Mg^2+^) ions present in the biofilm matrix support the cross-bridge formation, contributing to the polymer stabilization, and also facilitates the maturation and formation of biofilms to a thickness of around 300 µm (Sharma et al., 2023).

## The adherence of microorganisms to material surfaces

**Figure 2:**
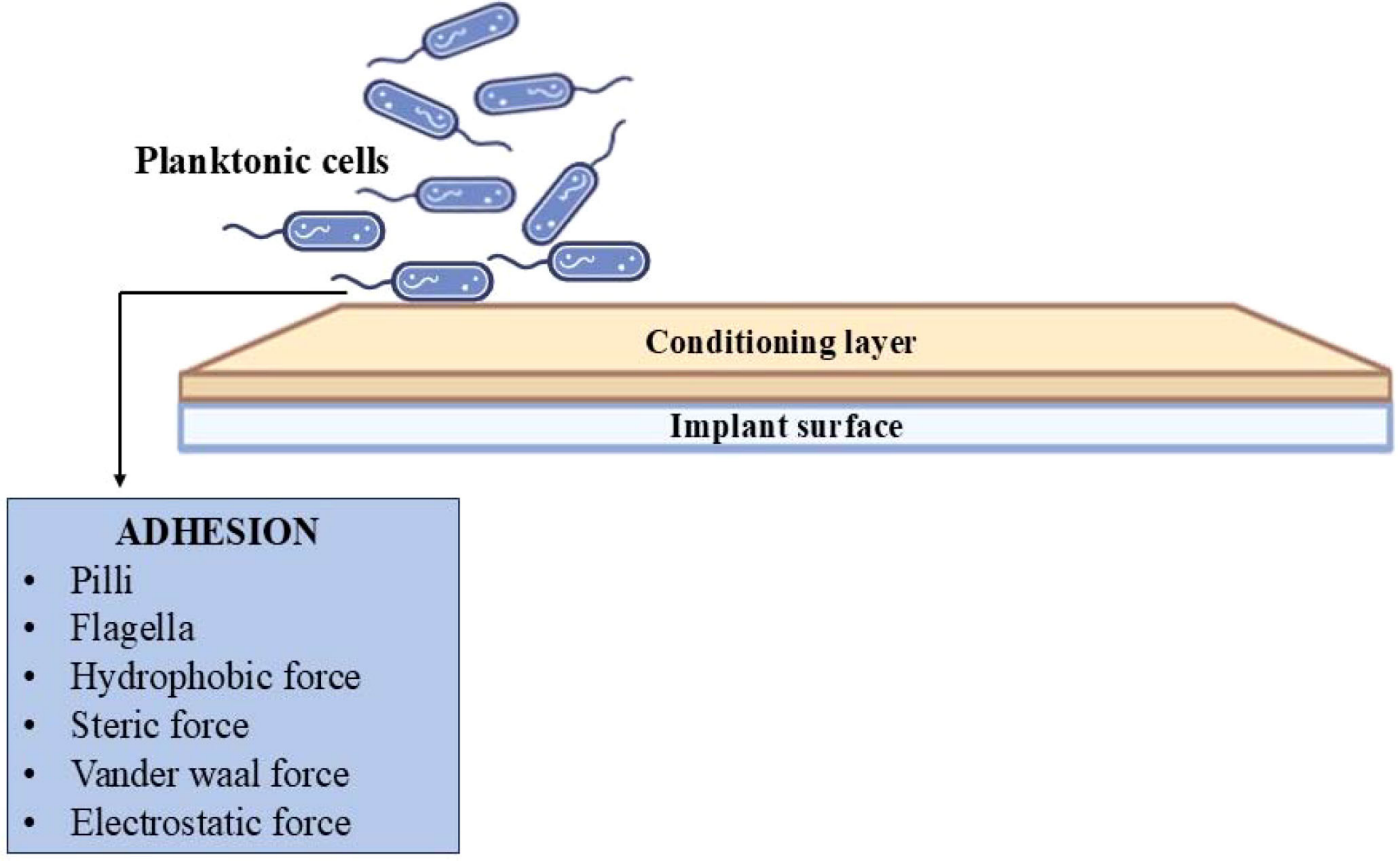
The image illustrates the visualization of bacteria adhering to the surface of an implant. The initial phase of biofilm development involves the adherence of planktonic cells to the implant surface. This adhesion process is a pivotal factor in the development of implant-associated infections, facilitated by structures like pili and flagella, along with forces such as hydrophobic, steric, van der Waals, and electrostatic interactions.

Bacterial adhesion is the process by which free-floating cells attach to the conditioning layer of implants with the help of adhesins (Khatoon et al., 2018; Filipović et al., 2020). Therefore, these bacteria utilizes flagella, pili, or various physical forces, such as steric, van der Waals, and hydrophobic forces, as well as protein adhesion, enhancing the attachment, as illustrated in **Figure 2**. Bacterial adhesion to a surface involves two phases mainly: an initial, immediate, and reversible physical phase, followed by a time-dependent, irreversible molecular and cellular phase (Ribeiro et al., 2012; Zhao et al., 2023). The factors influencing bacterial adhesion are outlined in **Table 5**.

**Table 5:** Key parameters influencing bacterial adhesion and surface colonization.

S.NO	Factor	Impact on adhesion	References
1	ENVIRONMENTAL FACTORS		
1a	Flow conditions	At a higher flow rate, decreased bacterial adhesion	(Ribeiro et al., 2012)
1b	Antibiotics	Decreased bacterial adhesion	(Ribeiro et al., 2012)
1c	Concentration of electrolytes (KCl, NaCl) and pH value	Cell survival rates rise with a slow increase in acidity.	(Ribeiro et al., 2012)
2	MATERIAL SURFACE CHARACTERISTICS		
2a	Surface chemistry	Bacterial adhesion depends on the functional group present in materials.	(Ribeiro et al., 2012)
2b	Surface roughness	Surface of irregular material promotes bacterial adhesion	(Ribeiro et al., 2012)
2c	Physical configuration	Porous materials have higher rate of bacterial infection	(Ribeiro et al., 2012)
2d	Surface free Energy	Higher surface free energy increases bacterial adhesion	(Yu et al., 2022)
3	BACTERIAL CHARACTERISTICS		
3a	Hydrophilicity	Hydrophilicity decreases, bacteria adhere to the surface by hydrophobic interactions	(Yu et al., 2022)
3b	Serum / tissue proteins (albumin, fibronectin)	Promote bacterial adhesion	(Ribeiro et al., 2012)
3c	Charge	Negatively charged bacteria are less likely get adhere to material with the same charge.	(Yu et al., 2022)

This table summarizes the key elements that promote bacterial adherence and colonization on various surfaces, particularly in relation to medical implants.

## The multistage process and constituent phases of biofilm formation

**Figure 3:**
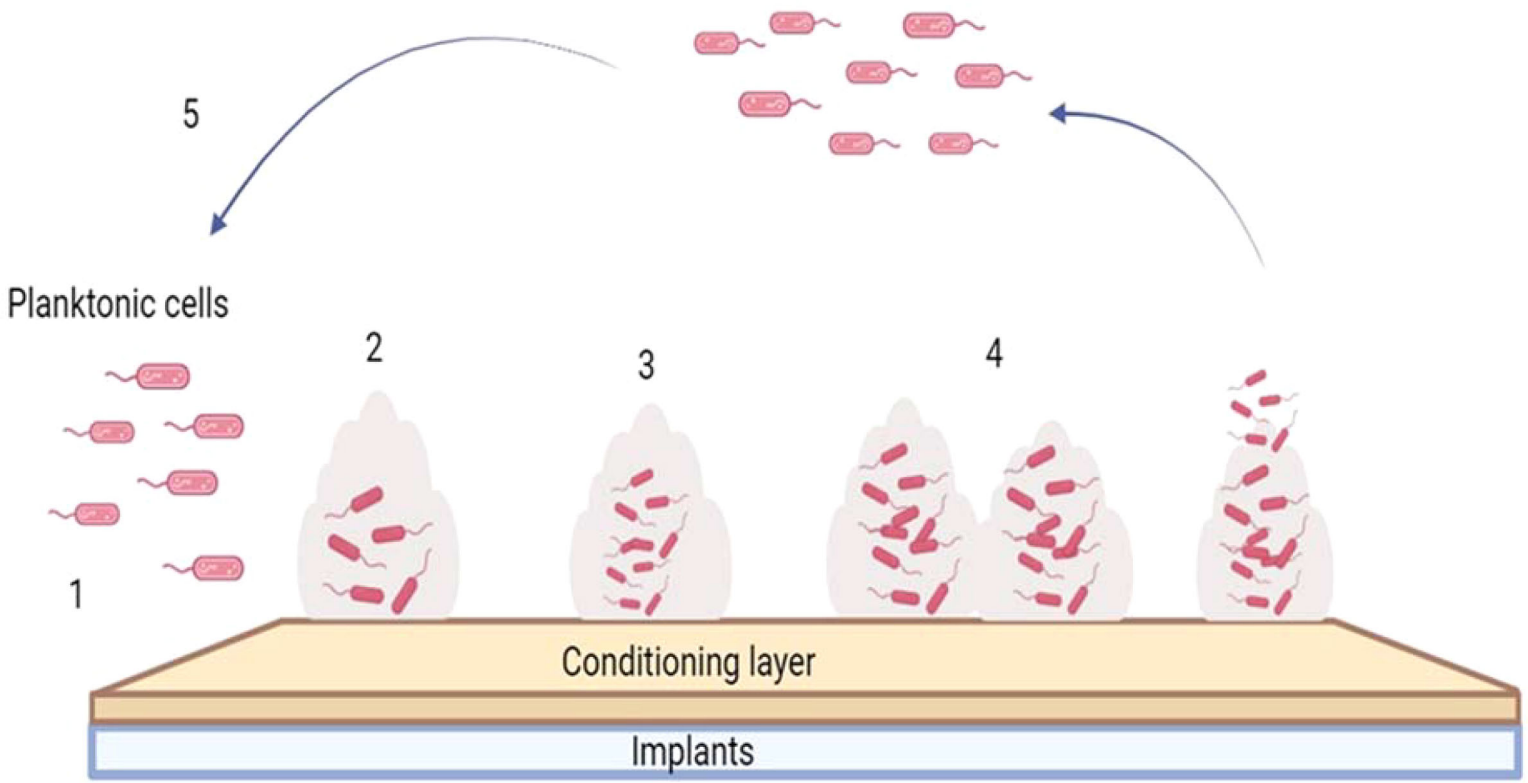
A schematic illustration outlining the various stages involved in biofilm formation on implant surfaces. The figure outlines the sequential stages of biofilm development on the implant surface: 1) adhesion of planktonic bacterial cells, 2) monolayer formation and production of matrix, 3) Microcolony formation with multilayer development, 4) maturation and proliferation into a biofilm, 5) detachment and dispersal of cells.

Biofilm formation involves a series of steps, as depicted in **Figure 3**. The process begins with adhesion, where free-floating cells (planktonic cells) attach to the conditioning layer on the implant surface using adhesins. After the bacterial adhesion onto the active site, the organism becomes sessile and begins to produce metabolites, thereby forming microcolonies as more cells assemble on the surface, building upon the initial monolayer. The EPS encases the bacterial cells, forming an organized structure called biofilm (Conen et al., 2017). The EPS acts as a structural framework, that enables bacterial attachment to the surface and provide nutrients and water. EPS is crucial in biofilm formation as they boost the bacterial resistance against several antimicrobial agents and protect bacteria from host immune defences. Biofilms become the major contributors to chronic infections on various indwelling medical implants, such as surgical implants and catheters. This can cause conditions including osteomyelitis, rhinosinusitis, cystic fibrosis, and wound infections (Singh et al., 2021). The attached cells mature and continue to grow, a process enhanced by the production of signaling molecules. Maturation involves two stages: Stage I is the interaction between cells and the production of autoinducer signal molecules, such as N-acylated homoserine lactone (AHL). Stage II consists of the establishment of a microcolony with a thickness and size of up to 100µm. After the maturation stage, the cells detach from the matrix, and the cycle continues. The biofilm matrix consists of DNA, teichoic acids, N-acetylglucosamine, host-derived products, and both dead and live cells. Bacterial cells exit the protective biofilm matrix during the dispersion stage, enabling their dissemination to other sites and leading to the spread of infection (Sauer et al., 2022; Sharma et al., 2023).

## The quorum-sensing process and its influence on biofilm development

Bacterial cells can sense their surroundings by detecting the production of auto-inducing signaling molecules through a process called quorum sensing. It is a bacterial process of intercellular communication facilitated by the production and detection of extracellular chemical signaling molecules called Autoinducers (AI). This process reduces the coordination of bacterial cells and their diverse responses to environmental stimuli. It occurs in both Gram-positive and Gram-negative bacterial species, enabling communication with one another, and promotes biofilm formation (Lahiri et al., 2021). The biofilm formation and quorum sensing is a closely connected phenomenon, where quorum sensing is a signaling process capable of governing the key stages of biofilm development. This includes initiation, matrix formation, maturation, and dispersal. It also coordinates collective phenotypes such as surface motility and extracellular polymeric substance production within the biofilm community (Lazar et al., 2021; Patil et al., 2021). The quorum-sensing gene initiates, promotes, and regulates the growth, maturation, and destruction of biofilms. N-Acyl homoserine lactone (AHL) is the key component of quorum sensing in Gram-negative bacteria whereas, in Gram-positive bacteria autoinducing peptides (AIP) promote biofilm formation (Shakibaie et al., 2015; Sharma et al., 2023). The studies indicates that ESKAPE pathogens activate the quorum-sensing pathways, including the LuxS system, which plays an important role in driving both antibiotic resistance and biofilm formation. This is because these bacteria depend on multiple quorum-sensing pathways to coordinate biofilm formation (Tiwari, 2023). **Table 6** outlines a comparative analysis of the quorum-sensing mechanisms employed by the ESKAPE pathogens, focusing primary signaling molecules.

**Table 6:** Quorum sensing in ESKAPE pathogens.

Pathogen	QS regulatory system	Signaling molecule	Role in biofilm	References
*Enterococcus faecium*	fsr system (FsrA/B/C), LuxS/autoinducer-2	Gelatinase, biosynthesis-activating pheromone (GBAP)	Controls production of gelatinase for biofilm formation, persistence of infection	(Ali et al., 2022; Santajit et al., 2022)
*Staphylococcus aureus*	agr system (Agr A/B/C/D),	Autoinducing peptide (AIP)	Expression of adhesins, toxins, and compounds that interfere with host immune responses	(Santajit et al., 2022; Yamazaki et al., 2024)
*Klebsiella pneumoniae*	LuxS	AI-2	Biofilm development, synthesis of lipopolysaccharides (LPS), capsule production, virulence	(Santajit et al., 2022; Bin Mohammad Muzaki et al., 2024)
*Acinetobacter baumannii*	LuxI/LuxR system, abaR/abaI	AHL	Surface motility, adhesion, aggregation, biofilm formation	(Zhong and He, 2021; Santajit et al., 2022)
*Pseudomonas aeruginosa*	Las (LasI/LasR), Rhl (RhlI/RhlR), and PQS	Signal ligands (3-oxo-C12-HSL and C4-HSL)	lectin, pyocyanin synthesis, and biofilm development.	(Santajit et al., 2022; Schlichter Kadosh et al., 2024)
*Enterobacter* sp.	LuxR	AI-1, AI-2, AI-3, C4-HSL and C6-HSLs	Adhesion, triggers QS-associated gene transcription, as well as its related phenotypes and biofilm formation	(Santajit et al., 2022)

This table provides a concise overview of the key quorum sensing systems identified within ESKAPE pathogens, emphasizing the signaling molecules involved and their functional roles in the modulation of virulence factors and biofilm development.

## The rising threat of ESKAPE pathogens and their biofilm-associated infections

The multidrug-resistant ESKAPE group of pathogens (also called superbugs), including *E. faecium, S. aureus, K. pneumoniae, A. baumannii, P. aeruginosa*, and *Enterobacter* sp., are capable of evading antibiotic treatments (Celebi et al., 2025). These bacteria adhere to various tissues and result in forming resilient biofilms in surgical sites, implants, and other devices. This becomes a significant issue in the prevention and management of infection (Lin et al., 2018). Infections due to biofilm can negatively affect various body parts, including teeth, lungs, skin, cardiovascular system, and urinary tract, with severe consequence. *S. aureus* is the chief causative agent of hospital-acquired pneumonia, while *P. aeruginosa* form biofilms within the lungs (Pugliese and Favero, 2002). Furthermore, multidrug-resistant Gram-negative bacterial species such as *E. coli, K. pneumoniae*, and *P. aeruginosa* are commonly associated with prevalent biofilm-based infections in healthcare settings (Antoci et al., 2008; Niveditha et al., 2012). Various microorganisms, including *P. aeruginosa, S. aureus, S. epidermidis, Serratia* sp.*, E. coli, Proteus* sp., and *Candida* sp., have been reported to adhere to and colonize contact lenses. Similarly, *E. faecalis, E. coli, S. epidermidis*, and *P. mirabilis* have been observed to colonize urinary catheters in the early stages of infection (Sharma et al., 2023).

*Enterococcus faecium*, a gram-positive and facultatively anaerobic bacterial species, is a known causative agent implicated in neonatal sepsis, meningitis, and other human infectious diseases (Motiwala et al., 2022). Vascular catheters or prosthetic implants can become infected by *E. faecium*, leading to prosthetic joint infections and surgical site infections. The increase in antibiotic resistance has made it difficult to remove the biofilm produced by this bacterium on the surface of implants. However, vancomycin, daptomycin, linezolid, and tigecycline commonly treat enterococcal infections (Holmberg and Rasmussen, 2014; Said et al., 2024).

*Staphylococcus aureus*, a gram-positive, facultatively anaerobic microorganism, is the most prevalent bacterial species that causes infections associated with medical implants (Motiwala et al., 2022). *Staphylococcus* is the most frequent bacterium that causes infections associated with implants (Zhang et al., 2014), with *S. aureus* and *S. epidermidis* being the two primary pathogenic strains. These bacteria commonly develop biofilm on various of medical devices, including central venous catheters, mechanical heart valves, pacemakers, intrauterine devices, and prosthetic joints (Tuon et al., 2023). The adhesion of these bacteria to the surface of implants and their subsequent formation of biofilms can lead to implant-associated infections (Götz, 2002; Vuong et al., 2004; Lu et al., 2022). *Staphylococcal* infections account for a large percentage of early postoperative prosthetic joint infections, at 57%, with *S. aureus* and *S. epidermidis* each accounting for 25% of cases. Furthermore, low pathogenic bacteria such as coagulase-negative *Staphylococcus* (61%), which have a high risk of *S. epidermidis* infections (35%), were the most common microorganisms responsible for late-chronic prosthetic joint infections (Lu et al., 2022). Treatments, including antibacterial coatings (silver nanoparticles), antiadhesive surfaces, and matrix-degrading enzymes such as Dispersin B and DNase I are used to prevent biofilm formation (Bhattacharya et al., 2015).

*Klebsiella pneumoniae*: Gram-negative, a facultative anaerobic organism capable of adhering to the surface of medical devices and causing pneumonia and urinary infections (Motiwala et al., 2022). Urinary catheters and inner walls of internal devices are mainly infected by *K. pneumoniae*, where bacterial colonization leads to urinary tract infections, respiratory infections, and gastrointestinal infections (Wang et al., 2020). The multidrug-resistant nature of bacteria causes several infections with high morbidity and mortality rates (Guerra et al., 2022). It is reported that the bacteria colonize the gastrointestinal tract, leading to the transmission of infection to other parts, and the multidrug resistance nature has become a major issue (Bouhrour et al., 2024). Incorporation of coating the implants with iron chelators and antagonising molecules along with bacteriophage approach over antibiotics can reduce the biofilm formation due to *K. pneumoniae* (Chhibber et al., 2013).

*Acinetobacter baumannii* Gram- negative aerobic bacterium mainly found in hospital environment causing chronic lungs and urinary tract infections (Motiwala et al., 2022). This bacterium is capable of producing biofilms on implants like urinary catheters and fracture fixation devices causing pneumonia, meningitis, wound infections, urinary tract infections and soft tissue infections (Morris et al., 2019; Bagińska et al., 2021; Kanakaris and Giannoudis, 2021). Therapeutic approaches like application of silver nanoparticles, antibacterial polymers and antibiotics like colistin, rifampicin, imipenem, and tigecycline can be employed to prevent colonisation of *A. baumannii*. Coating the implants with furanone, metal oxides and metal nanoparticles can inhibit bacterial adhesion in the implants (Upmanyu et al., 2022).

*Pseudomonas aeruginosa* is a gram-negative, facultative anaerobic bacterium that can cause urinary tract infections, joint infections, and respiratory tract infections (Magalhães et al., 2019; Motiwala et al., 2022). This bacterium has the potential to colonize medical devices, including. Studies show that *P. aeruginosa* is responsible for 28 % of IAI, and results in elevated mortality rates in infected patients due to its antibiotic resistance. Combined antibiotic therapy like cefepime-ciprofloxacin and ceftazidime-ciprofloxacin, can be used to manage bone and joint infections caused by *P. aeruginosa*. The other treatment techniques include photodynamic therapy, bacteriophage therapy, and application of enzymes (DNase I) can inhibit biofilm formation and quorum sensing (Cole et al., 2014; Cerioli et al., 2020; Kanakaris and Giannoudis, 2021; Yin et al., 2022; Akhmetzhan et al., 2023; Bouhrour et al., 2024).

*Enterobacter* sp. is a gram-negative, facultative anaerobic bacterium causing various infections like endocarditis, urinary tract infections, and soft tissue infections (Motiwala et al., 2022). *Enterobacter cloacae* were ranked as the tenth most common bacterium that causes nosocomial infections, and is known for producing biofilms on the implant surfaces (Misra et al., 2022). The *Enterobacter* is capable of infecting dental implants, leading to peri-implantitis (Nandakumar et al., 2013). *E. cloacae* and *E. aerogenes* can also form biofilms on heart valve implants and pacemakers, resulting in tissue injury, prosthetic valve endocarditis, valve degeneration, chronic inflammation, sepsis, pneumonia, and abscess formation. Introduction of new approaches, such as metal oxide materials, antimicrobial peptides, surface modification, and photodynamic treatment, helps to prevent bacterial adhesion and thereby inhibit biofilm formation on the implants (Kadirvelu et al., 2024).

## Methodologies for diagnosing infections related to medical implants

Bacteria can be recovered from implants by PBS washing, scalpel scraping, swabbing, and sonication. The implants are rinsed with PBS to remove debris and planktonic cells. The implants are then swabbed in selective media to identify the bacterial strain causing the infection. Compared to scraping with a scalpel, rinsing, and grinding, studies have shown that sonication is more effective at removing a higher quantity of bacterial biofilm from the implant surface (Kanakaris and Giannoudis, 2021).

**Figure 4:**
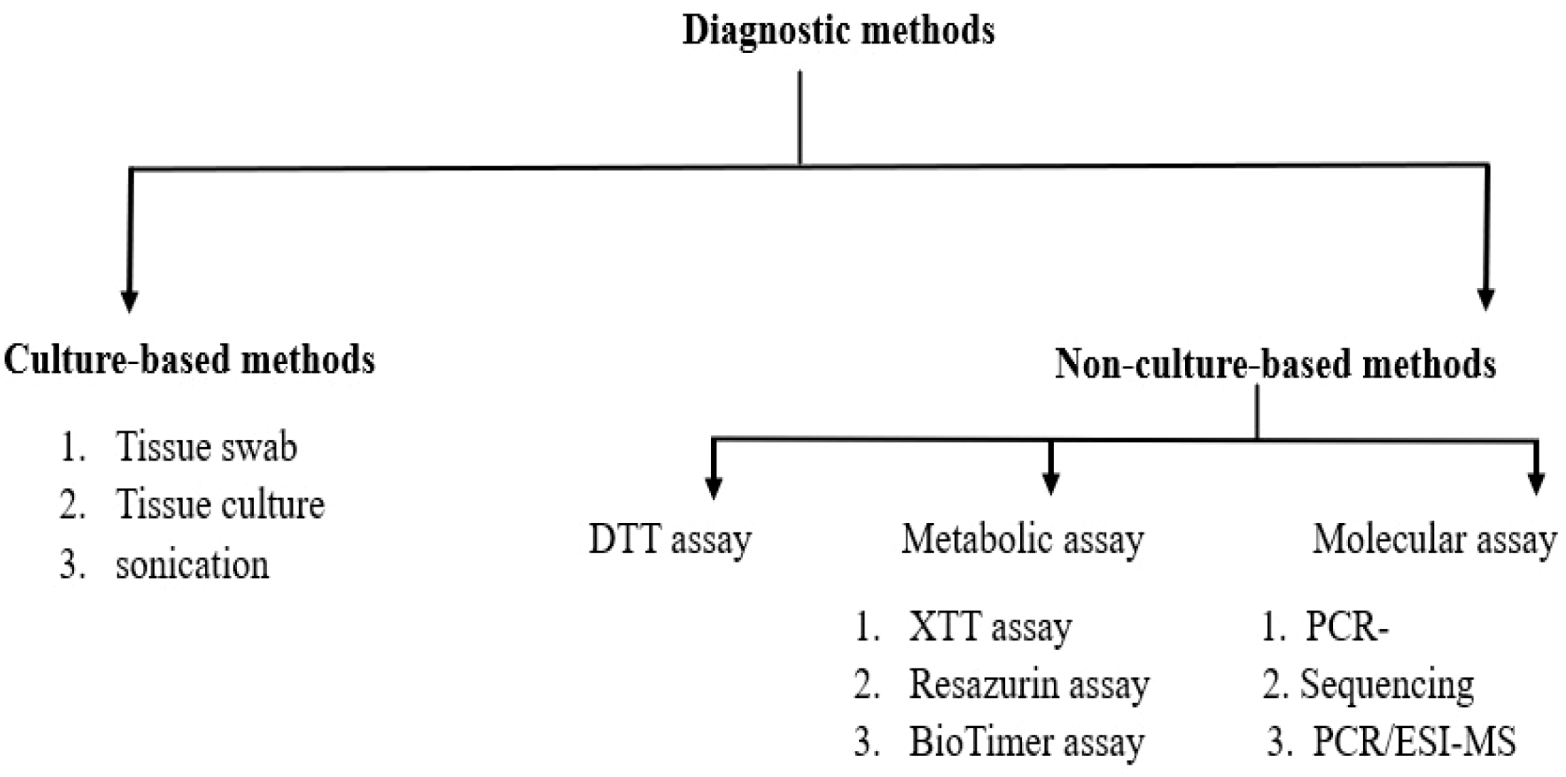
A figure outlining the various diagnostic techniques employed for detecting and identifying microorganisms. The presented figure elucidates diverse methodologies employed for the detection and characterization of pathogenic microorganisms implicated in implant-associated infections, encompassing both culture-dependent techniques, such as tissue swabbing, culturing, and sonication, alongside culture-independent approaches, including DTT assays, metabolic analyses, and molecular assays.

Bacterial fluid samples are further processed to diagnose associated infections. Various microscopic, culture-based, and non-culture-based techniques are used to identify the causative agents of implant-associated infections. **Figure 4**. provides an overview of the diagnostic techniques employed for the detection and identification of microorganisms. Common culture-based methods include tissue swabs, tissue cultures, and sonication. While tissue swabs and cultures can detect bacteria, but they are of low sensitivity. Sonication is preferred for diagnosing prosthetic joint and cardiac implantable electronic device infections, as it has higher sensitivity and can detect and enumerate bacteria. Non-culture-based techniques, such as assays and Polymerase chain reaction (PCR), (2,3-bis-(2-methoxy-4-nitro-5-sulfophenyl)-2H-tetrazolium-5-carboxanilide) XTT assay, and Resazurin assay, are also used to detect causative agents. Metabolic assays make use of specific indicators to measure the bacterial viability, while molecular tests, such as DNA/RNA extraction, PCR, and sequencing, are used for bacterial identification. Advances in next-generation sequencing (NGS) approaches, including 16S rRNA amplicon sequencing, metatranscriptomics, and shotgun metagenomics, have significantly enhanced bacterial identification. These new technologies are far more sensitive than regular culturing methods, but their scope remains constrained by the specificity of the employed primer sets. Mass spectrometry techniques are used to evaluate bacterial protein profiles. At the same time, several microscopic techniques like light microscopy, scanning electron microscopy, fluorescence microscopy, and confocal laser scanning microscopy are employed for to detect IAI. To diagnose prosthetic joint infections, gram staining is performed along with light microscopy but due to their low sensitivity makes the technique less reliable (Tande and Patel, 2014; Azad and Patel, 2024; Giarritiello et al., 2024). **Table 7** is an overview of various detections methods employed for identifying infections in medical devices.

**Table 7:** Detection methods for implant-associated infections in medical devices.

Implant type	Detection method	References
Orthopedic implants	Histopathology studies Culture-dependent methods (tissue collection, sonication) Culture-independent methods (PCR, NGS)	(Ponraj et al., 2021)
Dental implants	Fluorescence-based approach	(Hwang et al., 2021)
Sutures	Swab culture (Levine technique) Needle aspiration Tissue biopsy Telemedicine	(Li S. et al., 2021; Lathan et al., 2022)
Urinary catheters	Microscopy (confocal laser scanning microscopy, fluorescence microscopy)	(Oliva et al., 2018)
Cardiac devices	Sonication method Amplicon-based metagenomic approaches (NGS)	(Oliva et al., 2018; Olsen et al., 2022)
Breast implants	Scintigraphy Periprosthetic fluid diagnosis	(Stewart et al., 2020; Giovannini et al., 2023)
Cochlear implants	Transcutaneous ultrasound	(Rupp et al., 2022)

This table elucidates a comprehensive array of diagnostic modalities employed in the detection of infections associated with medical implants, encompassing a variety of device types. It includes both conventional and advanced approaches, highlighting their detection methods across different implants.

## Detrimental effects of biofilm development

Once bacteria adhere to the surface of implants, they begin to colonize and form biofilm. This is because the bacterial load required to contaminate implants is comparatively lower than that needed for native tissues. The biofilm formed on implant surfaces can cause serious health conditions in patients, including cystic fibrosis, periodontitis, otitis media, chronic bacterial prostatitis, non-healing infected chronic wounds, kidney infections, meningitis, and chronic sinusitis, resulting in implant failure (Khatoon et al., 2018; Shineh et al., 2023). The production of certain toxins and enzymes by the bacterial biofilm can cause damage to host tissue and delay the healing process (Mendhe et al., 2023).

**Figure 5:**
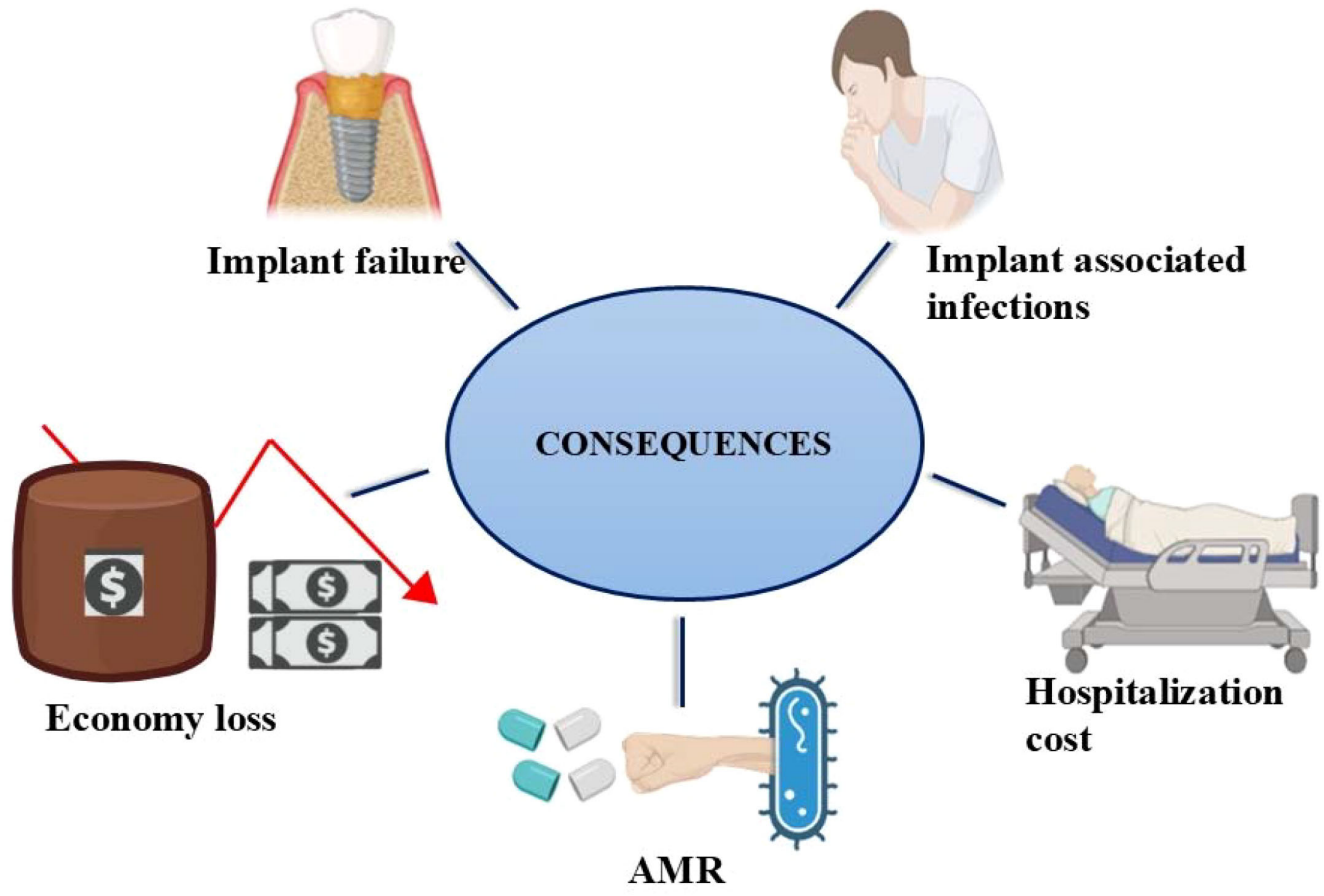
A visual depiction of the implications of biofilm formation. The formation of biofilms precipitates major adverse outcomes, including elevated antimicrobial resistance, implant failure, implant-associated infections, increased hospitalization costs, and a significant economic burden.

On a global scale, the economic loss due to urinary tract infections, infective endocarditis, and wound infections is estimated to be approximately $1 billion, $16 billion, and $281 billion per year, respectively (Shineh et al., 2023). The biofilm formation on the implant surface reduces their performances and eventually causes implant failure (Nandakumar et al., 2013). Implant failure due to biofilm can impose significant financial burdens on healthcare systems and patients, including high hospitalization costs, and repeated surgeries that ultimately result in death or a decrease in quality of life, as depicted in **Figure 5** (Veerachamy et al., 2014).

## Biofilm enhanced antibiotic resistance

Biofilm possess a protective layer called exopolysaccharide matrix, which helps the bacteria from antimicrobial agents making them more resistant to traditional cleaning methods. Furthermore, this matrix can inhibit antibiotic penetration, reducing the drug’s ability to eradicate the bacteria. Biofilm-associated infections account for approximately 80% of all infections, and treatment with antibiotics has become increasingly ineffective due to the microorganisms’ acquired resistance. Increasing the antibiotic dosage is not recommended due to adverse effects, and replacing implants is impractical because of the high costs. Treatment is further complicated by the diverse microorganisms present within the biofilm (Esteves et al., 2022; Mendhe et al., 2023). Bacteria become resistant to antibiotics due to several reasons, such as hypoxic conditions, decreased growth, physiological variability, oxidative stress responses, efflux pumps, quorum sensing, persister cells, horizontal gene transfer, as limited drug penetration, the presence of antibiotic-modifying enzymes, and elevated mutation rates (Rather et al., 2021; Ciofu et al., 2022). The higher concentration of DNA and close proximity of cells in biofilm enhance horizontal gene transfer, leading to increased antibiotic resistance (Nandakumar et al., 2013). ESKAPE pathogens have acquired genetic mechanisms that confer resistance to a broad range of antibiotics, including oxazolidinones, lipopeptides, macrolides, fluoroquinolones, tetracyclines, β-lactams, and β-lactam–β-lactamase inhibitor combinations. The bacteria gain resistance through genetic mutations and the acquisition of mobile genetic elements (De Oliveira et al., 2020). Consequently, remediation of infected implants through replacement or cleaning proves ineffective, necessitating the development of more effective techniques to mitigate bacterial adhesion and biofilm formation on implants, thereby reducing the incidence of IAI.

## Approaches to regulate and manage bacterial biofilm formation on implants

Biofilm control is crucial for reducing infections and increasing mortality rates, as antibiotic-resistant bacteria emerge due to the frequent use of antibiotics. Enzymes like DNase, proteinase K, and trypsin can degrade the matrix proteins and extracellular DNA components within the *S. epidermidis* biofilm, which in turn diminishes the structural integrity and resilience of the biofilm (Roy et al., 2018). By altering the surface to reduce adhesive properties or creating antibacterial coatings, numerous initiatives have been undertaken to combat the adherence of bacteria to implants (Filipović et al., 2020; Lu et al., 2022). Research has demonstrated that using a group of monoclonal antibodies to treat biofilms has decreased their formation and prevented biofilm-related infections. Studies have demonstrated that monoclonal antibodies 12C6, 12A1, and 3C1 can inhibit the growth and reduce the adherence of the bacterial accumulation-associated protein in *S. epidermidis* (Freire et al., 2017). Bacteriostatic or bactericidal agents, like vancomycin, are employed to inhibit the attachment of bacteria and the subsequent formation of biofilms on the surfaces of medical devices, such as metal implants. Research has shown that *S. epidermidis* biofilm formation is inhibited, while another research has shown that bacteria develop vancomycin resistance. These findings provide evidence both in favor of and against the technique of using vancomycin. Implant coatings change the surface chemistry or structure to decrease bacterial adherence. They can be passive or active. To lessen infection, active coatings emit preservatives and antibiotics, among other bactericidal agents. However, because of the quick coverage of plasma proteins, these approaches might not work (Sharma et al., 2023).

The hydrophilic nature of polyethylene glycol (PEG) coatings creates a hydration layer that keeps bacteria at bay, making them popular anti-adhesive coatings. PEG is a less effective choice because, although chemically stable, it is readily oxidized and does not offer long-term antibacterial protection after implantation. Many coatings can also prevent germs from adhering to them by blocking their hydration layers, including hyaluronic acid coatings, chitosan coatings, and zwitterionic polymer coatings. Zwitterionic polymers are among them and have drawn a lot of interest lately. Moreover, UV-irradiated titanium dioxide has antibacterial properties and is very hydrophilic. Due to its remarkable properties, such as high hardness, low friction coefficient, chemical inertness, and corrosion resistance, titanium nitride can also impede bacterial adhesion, thus reducing their interactions (Maddikeri et al., 2008; Lu et al., 2022).

Tissue plasminogen activator (tPA), one type of fibrinolytic coating, can lessen the *in vivo* and *in vitro* production of *S. aureus* biofilms on medical equipment. Because these coatings break local fibrin, they inhibit early bacterial adhesion and biomass buildup. They also make biofilm infections more susceptible to the effects of antibiotics (Kwiecinski et al., 2016). The application of oxygen plasma coatings with monomeric trimethylsilane results in surface chemical modifications that decrease bacterial adherence and prevent the development of biofilms. To stop staphylococcal binding and consequent infections, biomaterial surfaces might be coated with direct thrombin inhibitors (Li X. et al., 2021).

Numerous pathogens have been used to demonstrate the antibacterial and antibiofilm properties of nanoparticles (NPs). The effectiveness of antibiofilm nanomaterials depends on various characteristics, including the size, shape, surface charge, structure, material composition, and concentration of the particles. Examples of such nanomaterials include inorganic materials like gold and silver (Mohanta et al., 2020; Franzolin et al., 2022), polymers such as chitosan (AL-Fawares et al., 2024), lipids like liposomes, and molecular complexes comprising proteins and cyclodextrin. Metal nanoparticles have been shown to have an influence on biofilms and to be efficient bactericides against a variety of bacteria. As such, they provide a promising approach to the prevention of biofilm formation. According to a recent study, chitosan nanoparticles (CNPs) can destroy mature biofilms, prevent the creation of new *S. aureus* biofilms, lessen EPS synthesis, and decrease the hydrophobicity of the cell surface (Li X. et al., 2021; Ghosh and De, 2023).

Despite the effectiveness of silver nanoparticles in preventing biofilm formation on medical equipment, excessive exposure to silver can harm human cells. Similarly, the antimicrobial furanone coating has been shown to inhibit *S. epidermidis* biofilm growth (Hume et al., 2004). AgNPs, SeNPs, and ZnO NPs are among the nanoparticles that have anti-quorum sensing action against ESKAPE pathogens. They inhibit the synthesis of AHL, downregulate luxA and luxR, and lessen the release of several virulence agents that the quorum sensing system mediates. CuO, ZnO, MgO, TiO_2_, Al_2_O_3_, and Fe_3_O_4_ are examples of metal oxide nanoparticles that exhibit biofilm inhibition and disruption capabilities. These nanoparticles release their metal ions causing physical damage to the cell wall, disrupt cellular functions and produce oxidative stress by generating reactive oxygen species (Mukherjee et al., 2023).

Natural products, especially herbal compounds are gaining more attention due to their strong antibiofilm activity, lower toxicity, and safety over a wide range of bacteria. Curcumin extracted from *Curcuma longa* prevents quorum sensing by interacting to LasR and LuxR receptors in *P. aeruginosa*. Studies with allicin, from *Allium sativum L*. reported for its potential in suppressing bacterial adhesion by inhibiting the formation of EPS. The compound baicalin, from *Scutellaria baicalensis* in *S. aureus*, had showed significant results in suppressing quorum sensing and inhibits from producing autoinducers (Mishra et al., 2020; Zhang et al., 2022). Essential oils are reported for their ability to manage IAI. The *in vitro* studies demonstrated that essential oil obtained from *M. longifolia* is potent against both Gram-positive and Gram- negative bacteria growing on implant surfaces of titanium and steel (Pazarci et al., 2019). The biofilm-associated infection in the skin-implant interface was found to be reduced due to development of essential oil-based oregano gel formulation which exhibited strong antibiofilm activity against *S. aureus* (Ong et al., 2022). The main limitations of these compounds are that they function slowly, effective only at higher doses, and fails to kill bacteria directly. However, their efficacy can be increased when coupled with an antibiotic therapy (Zhang et al., 2022).

**Figure 6:**
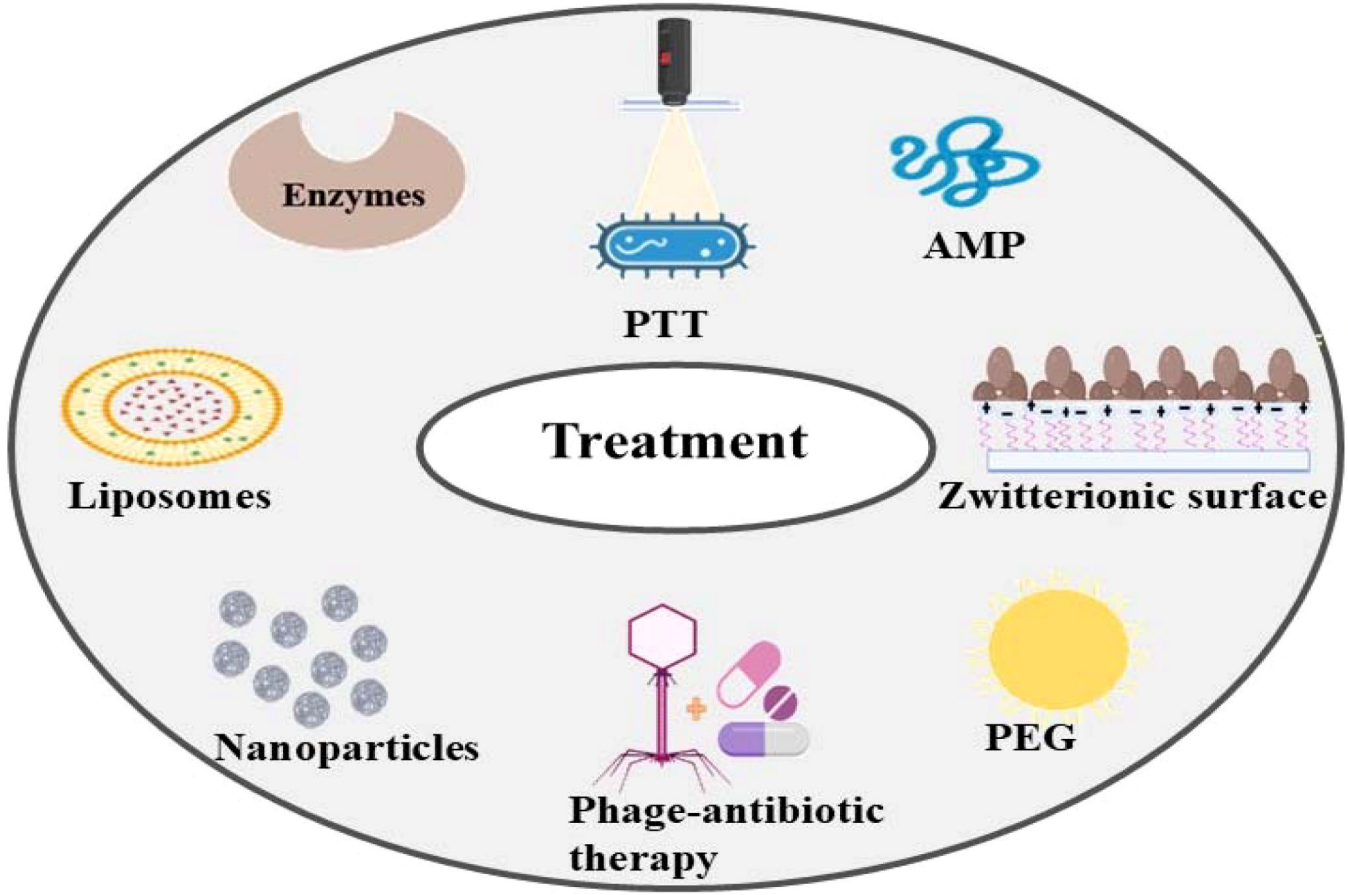
Therapeutic interventions for addressing biofilm infections. The illustration presents a variety of strategies designed to combat biofilm-mediated infections. These strategies include the use of enzymes, liposomes, nanoparticles, PEG-based coatings, phage-antibiotic therapy, photothermal therapy (PTT), zwitterionic surfaces, and antimicrobial peptides (AMP), each contributing to the prevention or disruption of biofilm formation.

The selection of the coating agents is greatly influenced by the surface properties. Based upon the existing literature, only limited studies have been conducted on the persistent bacteria even though the nanoparticles are proved for their antibiofilm activity. To eradicate the persistent cells, a new technology has evolved that make use of advanced nanoparticles with antibiofilm techniques. These include photothermal therapy (PTT) and photodynamic therapy (PDT) which can be effective in reducing the persistent bacteria. In, PTT, near-infrared light gets converted into local heat using photo thermal agents causing destruction of bacteria and biofilm structure thereby enhancing the distribution of photosensitising agent. This method is limited as it requires higher temperature to kill the bacteria effectively (Huo et al., 2021; Lu et al., 2022). The algal compounds are proved for their strong antibiofilm activity but due to their toxicity, resistance, higher degradation chance and concentration requirement makes the treatment ineffective (Khatoon et al., 2018; Esteves et al., 2022; Mukherjee et al., 2023). An overview of various treatment methods to reduce biofilm formation is showed in **Figure 6**

Therefore, to overcome these limitations, future research should emphasise on efficient removal of biofilms, reduced toxicity and antimicrobial resistance making a way to develop cost effective and advanced treatment approaches.

## Challenges in treatment

Biofilm has become a major concern in the medical sector, due to the bacterial adhesion causing implant failure and associated infections. The antimicrobial resistance provided by EPS that forms biofilm on the implants can be the main barrier in treating biofilm-associated infections (Mah and O’toole, 2001; Venkatesan and Sikkander, 2023). The chronic nature of biofilm-associated infections and their complex nature of biofilm make the treatment extremely difficult (Bjarnsholt et al., 2008). The heterogeneous nature of biofilm and its microbial composition make the treatment difficult (Flemming et al., 2016). Surface roughness and energy help the bacteria to promote biofilm formation in implants (Franco et al., 2023; Sinjab et al., 2024), and modifying the surface of the implant to prevent bacteria remains a difficult task. As a result, research should aim to develop therapies that prevent biofilm formation on implant surfaces, to overcome antibiotic resistance, improve implant biocompatibility, and enhance medical care. In turn, this study can improve patient quality of life, decrease the incidence of implant-associated infections, and lower medical expenses.

## The incorporation of anti-biofilm coatings on medical implants

To prevent biofilm formation on implants, these devices are coated with various antibiofilm drugs using techniques such as physical adsorption, sol-gel, layer-by-layer deposition and chemical vapor deposition (Shahid et al., 2021; Ul Haq and Krukiewicz, 2023). The different approaches to incorporating antibiofilm coatings on implants are outlined in the **Table 8**. These antibiofilm coating are designed in a way to achieve its goals such as inhibition of bacterial adhesion and to hinder the initial stages of biofilm formation, helping in the management of IAI, thereby enlightening the quality of life in the patients.

**Table 8:** Antibiofilm coating strategies.

S.No	Coating method	Mechanism	Benefits	Challenges	Reference
1.	Furanone-releasing coatings	Coating kills the attached bacteria	Interferes with quorum-sensing pathways, prevents initial bacterial attachment of gram-positive and gram-negative bacteria	Lesser data on cell viability and toxicity studies	(Cheng et al., 2012; Shahid et al., 2021)
2.	Antimicrobial Peptides (AMP)	Disrupts cell membrane and eliminates the bacteria	Broad spectrum activity, biocompatible, low chance of developing resistance and toxicity.	High cost, degradation by host	(Shahid et al., 2021; Esteves et al., 2022)
3.	Silver nanoparticles (AgNPs) using sol-gel technique	Generate reactive oxygen species which suppress growth and eliminate bacteria.	Potent, wider antibacterial spectrum	Cytotoxic to eukaryotic cells, decreased osseointegration property of implants.	(Esteves et al., 2022; Ul Haq and Krukiewicz, 2023)
4.	Antibiotic coating- sol gel technique	Antibiotics are directly administered into implant site and destroys the pathogen	cost-effective, broader antibacterial spectrum, higher efficacy	Antibiotic resistance, toxicity, short term efficacy	(Shahid et al., 2021; Esteves et al., 2022)
5.	Polymeric coating	Coating releases drug in controlled manner preventing biofilm	Biocompatibility, drug delivery vehicles	Adhesion between coating and substrate is not strong	(Saberi et al., 2021; Valliammai et al., 2021; Ul Haq and Krukiewicz, 2023)
6.	Smart material coating	Coating releases loaded antimicrobial in response to bacterial growth	Controlled drug release, good cytocompatibility	Expensive and complex to manufacture	(Ul Haq and Krukiewicz, 2023)
7.	2D-Nanomaterial-Based Nanocoating (Graphene, black phosphorus)	Coating destructs cell wall and kills the bacteria	Biocompatibility, easy-to-fabricate, low cytotoxicity	Long-term effects are unknown.	(Sahoo et al., 2022)
8.	Nitric oxide-releasing coatings using sol gel technique	Release nitric oxide and prevents adhesion	bactericidal effect, low concentration required	Short lived and low diffusion distance	(Rao et al., 2020; Shahid et al., 2021)
9.	Polyethyleneimine (PEI); synthetic cationic polymers	Coating destroys the cell membrane	Low-cost and commercially available	High cytotoxicity, low biocompatibility	(Shahid et al., 2021)

This table is an overview of different coating techniques, their mechanisms, benefits and challenges to eradicate the biofilm formation.

## Prospective future directions

The emergence of multidrug-resistant ESKAPE pathogens has greatly hindered the treatment of implant-associated infections, largely because they can form resilient biofilms. These biofilms formed on the surface of implants will shield the bacteria from various antibiotics making them more resistant towards the host immune system. Beyond ESKAPE pathogens, there are several other pathogens irrespective of their resistance, forms biofilms marking extreme difficulty in removing them due to their inbuilt tolerance. However, additional characteristics of ESKAPE pathogens such as multidrug resistance, EPS production, and quorum sensing often makes the treatment of biofilm-related infections more challenging (Santajit and Indrawattana, 2016; Santajit et al., 2022).

Recent studies show rifampin, an antibiotic exhibiting higher antibiofilm activity towards staphylococcal infections in implants. However, long-term use of rifampin can cause adverse effects, including gastrointestinal complications and hepatotoxicity, and its effectiveness can be variable in the presence of implants (Zimmerli and Sendi, 2019; Renz et al., 2021). Conversely, fluoroquinolones are effective against Gram-negative bacilli, but many Gram-negative ESKAPE pathogens have developed resistance to these antibiotics. In fact, fluoroquinolones are struggling to effectively clear urinary tract infections in up to 50% of cases, with common fluoroquinolones like levofloxacin and ciprofloxacin exhibiting reduced response rates against Gram-negative bacteria, including *E. coli, K. pneumoniae, A. baumannii, and P. aeruginosa*. Mutations in target enzymes, efflux pumps, porins, and plasmid-mediated mechanisms primarily drive fluoroquinolone resistance in Gram-negative bacteria. To overcome the resistance mechanisms in fluoroquinolone antibiotics, antibiotic resistance breakers (ARBs) can be utilized, either directly conjugated to the fluoroquinolone molecule or administered in combination (Kherroubi et al., 2024).

Despite the promising results from animal studies of antimicrobial coatings, they are not yet widely applicable due to the lack of sufficient long-term data on their effectiveness and the growing problem of multidrug-resistant pathogens. Developing alternative antibiofilm coatings antibiofilm coating for implants is a crucial area of research. Further research should focus on hydrophobic, smart material and microbicidal polymer coatings, as these can overcome challenges and promote long-term effectiveness in eliminating pathogens (Shahid et al., 2021). Therefore, further research should be carried out giving more attention on the following areas such as designing personalised implants that help each patient to meet their requirements, enhancing the adhesion between coating and the implant surface, which can increase the mechanical stability and durability of the coatings (Negut et al., 2024). These enhancements can lead to the evolution of safer and more long-lasting medical implants, which would reduce the global issue of infections caused by ESKAPE pathogens.

## Conclusion

The review provided an overview of IAI, with a major focus on biofilm-forming ESKAPE pathogens, and described various methods for bacterial diagnosis, including current approaches to prevent infection in the implants. The methods discussed provide a variety of *in vitro* approaches to investigate the interactions between bacteria and materials. Each approach has advantages and disadvantages, but they collectively contribute to our understanding of these interactions, despite the expense, complexity, and time required to obtain findings. Further scientific and clinical investigation on pathogens infecting implants is urgently needed, as biofilm-associated implant infection remains a serious public health issue. It is necessary to give more focus to the *in vivo* assessment of anti-biofilm therapeutics, and additional research is required to develop new implant surfaces that inhibit bacterial adherence over the implant surface. Despite the availability of *in vivo* models, many studies lack pharmacokinetic and pharmacodynamic data, and high synthesis costs further limit the clinical translation of antibiofilm peptides (Silveira et al., 2021). In addition, limitations in using the vertebrate model for studying implant-associated infection have restricted experimental design, thereby limiting translation to human health due to ethical concerns, the need for long-term monitoring, and procedures that cause significant stress and pain in animals. Therefore, introduction of the 3R principle, such as replacement, reduction refinement) can be adopted as an alternative model to address these limitations (Mirza et al., 2025). Therefore, developing efficient, safe, and biocompatible implants that can limit biofilm formation is essential to prevent implant-associated infections and subsequent implant failure.
